# Association of functional competencies with vaccination among older adults: a JAGES cross-sectional study

**DOI:** 10.1038/s41598-022-22192-2

**Published:** 2022-10-14

**Authors:** Kousuke Iwai-Saito, Koryu Sato, Katsunori Kondo

**Affiliations:** 1 Division of International Health, Graduate School of Medical and Dental Sciences, Niigata University, Niigata, Japan; 2 Department of Social Epidemiology, Graduate School of Medicine and School of Public Health, Kyoto University, Kyoto, Japan; 3 Department of Social Preventive Medical Sciences, Center for Preventive Medical Sciences, Chiba University, 1-8-1, Inohana, Chuo-Ku, Chiba 360-0856 Japan; 4 Department of Gerontological Evaluation, Center for Gerontology and Social Science, Research Institution, National Center for Geriatrics and Gerontology, Aichi, Japan

**Keywords:** Public health, Epidemiology, Preventive medicine

## Abstract

It is unknown whether higher functions in sublevels of competence other than instrumental activities of daily living (IADL) are associated with vaccinations. This study examined whether higher functions, including intellectual activity (IA) and social role (SR), were associated with vaccinations among 26,177 older adults. Older adults with incapable activities in IA and SR had increased risks for non-receipt of influenza vaccinations (IA: for one incapable task/activity: incident rate ratio (IRR) = 1.05, 95% confidence interval (CI) = 1.02–1.09; SR: for two incapable tasks: IRR = 1.12, 95% CI = 1.08–1.16). Those with incapable activities in IADL and IA had increased risks for non-receipt of pneumococcal vaccination (IADL: for two incapable tasks: IRR = 1.13, 95% CI = 1.05–1.23; IA: for two incapable tasks: IRR = 1.10, 95% CI = 1.08–1.12). Those with incapable activities in IADL, IA, and SR had increased risks for non-receipt of both of the two vaccinations (IADL: for two incapable tasks: IRR = 1.17, 95% CI = 1.03–1.33; IA: for two incapable tasks: IRR = 1.18, 95% CI = 1.11–1.25; SR: for two incapable tasks: IRR = 1.13, 95% CI = 1.07–1.20). Having a family physician mitigated associations for non-receipt, regardless of competency. Our results suggest—maintaining the higher functions are important for older adults to undergo recommended vaccinations as scheduled; also, having a family physician to promote vaccinations is beneficial even for older adults with limited functions.

## Introduction

Influenza and pneumonia become more severe or lethal in older adults aged 65 years compared to younger adults. Human influenza viruses A and B typically cause mild respiratory illness and infection of the lower respiratory tract, which can progress to pneumonia, acute respiratory distress syndrome or secondary bacterial infection, and death from respiratory failure^1^. An average of 389,000 respiratory deaths were estimated to be associated with influenza worldwide each year during the study period, or ~ 2% of all annual respiratory deaths and 67% of these were among older adults aged ≥ 65 years^2^. Pneumonia is a major cause of mortality and morbidity among older adults. *Streptococcus pneumoniae* is a leading cause of morbidity and mortality of the illness globally, contributing to 1,189,937 deaths, which is more than all other etiologies^3^

Influenza and pneumococcal vaccinations are effective in preventing older adults from these severe illnesses^4,5^. However, social, or geriatric factors may hamper them from receiving vaccinations. For example, living alone, paucity of social capital^6–8^, and cognitive impairment can be risk factors for non-receipt of vaccinations among older adults^9^. The lower capability of instrumental activity of daily living (IADL) is one such geriatric factor among older adults. IADL is a measure for assessing “instrumental self-maintenance,” which was defined as one of Lawton’s seven sublevels of competence, which include life maintenance, functional health, perception and cognition, physical self-maintenance, effectance, and social role (SR)^10^. Manias et al. recently reported that the lower capability in IADL was associated with the lower uptake of influenza and pneumococcal vaccinations among older adults aged 65 years and more 3 months after discharged from geriatric rehabilitation^11^. However, it is unknown whether higher-level functions of competency other than IADL, such as intellectual activity (IA) and SR, are associated with influenza and pneumococcal vaccination uptake in older adults. Examining these associations is important because higher-level functions are more likely to be lost as aging than IADL^12^.

As a result, we evaluated whether lower capabilities of the higher functions, including IA and SR, were associated with non-receipt of influenza and pneumococcal vaccinations among community-dwelling older adults aged 65 years or more. Furthermore, we hypothesized that having a family physician could mitigate the relationship between competency deterioration and non-receipt of the vaccinations and assessed its effect modification^13^.

## Methods

### Study design and participants

This study had a cross-sectional design and used data from the Japan Gerontological Evaluation Study (JAGES). In Japan, this study looked at the social determinants of health among non-institutionalized and functionally independent people aged 65 years and more who did not require assistance from public long-term care insurance. We mailed self-reported questionnaires to older adults in 64 municipalities from November 2019 to January 2020. We invited 45,974 individuals, and 31,495 returned the questionnaire, corresponding to a response rate of 68.5%. We excluded 5,318 people from the study because they did not agree with the academic consent, were under the age of 65 years, or did not provide information about their age or sex. As a result, the analytical data consisted of 26,177 individuals.

### Outcome variables

The outcome variable was non-receipt of influenza, pneumococcal, or the both vaccinations according to the recommended schedule^14–16^. We examined the three outcomes separately because our recent study suggested heterogeneous determinants of the vaccinations^13^ and we found that the participants who received neither of the two vaccinations were prevalent among all the participants (24.1%, Supplemental Table 4). We asked, “Did you get an influenza vaccination in the last year?” and “Did you get a pneumococcal vaccination in the last 5 years?” We created binary variables for influenza, pneumococcal, or both of the two vaccinations 1 indicating those who did not undergo the vaccination and 0 indicating otherwise.

### Explanatory variables

Explanatory variables were the incapability of IADL, IA, and SR, measured using the Tokyo Metropolitan Institute of Gerontology Index of Competence (TMIG-IC)^17^. The IADL was assessed by asking the participants, “Can you go out alone by train or bus?” “Can you go shopping for daily necessities?” “Can you cook for yourself?” “Can you pay your bills by yourself?” “Can you deposit or withdraw money from your bank/postal savings account(s) by yourself?” For each question, we assigned 1 for those who answered, “No, I can’t,” and 0 for those who answered, “Yes, I can and do,” or “Yes, I can but usually don’t.” We added the number of incapable activities and categorized it into 0, 1, 2, and more. IA was assessed by asking participants, “Can you complete paperwork for your pension or other reasons (e.g., documents submitted to your local government offices, clinics/hospitals, etc.) by yourself?” “Do you read newspapers?” “Do you read books or magazines?” “Are you interested in health-related articles or TV programs?” SR was assessed by asking participants, “Do you visit your friends’ homes?” “Do you advise your family members or friends?” “Can you visit people who have fallen ill?” “Do you start conversations with young people?” For IA and SR, there were yes/no options. We assigned 1 for the response of “No” and 0 for “Yes” and categorized the number of incapable activities for IA and SR into 0, 1, 2 and more. In addition, the total number of incapable activities in the TMIG-IC (i.e., the sum of IADL, IA, and SR) was also categorized into 0, 1, 2, and more. For IADL, IA, SR, and the TMIG-IC, we set 0 (i.e., fully capable) for the reference group.

### Covariates

For individual covariates, sociodemographic data, behavior, health status, and social relationship were used. Sociodemographic covariates consisted of sex, age (65–74 and ≥ 75 years), marital status (married, widowed, divorced, never married, and others), educational attainment (< 6, 6–9, 10–12, ≥ 13 years, and others), equivalized income (< 0.5, 0.50–0.99, 1.00–1.99, 2.00–3.99, and ≥ 4.00 million yen), and household structure (living alone, living with a spouse, living with offspring, living with a spouse and offspring, living in a three-generation household, and others)^18^. Behavioral covariates consisted of smoking status (never smoked, quit smoking ≥ 5 years ago, quit smoking < 5 years ago, smoke sometimes, and smoke almost every day), self-rated health (excellent, good, fair, and poor), having a medical checkup (within 1 year, sometimes between 1 and 4 years ago, > 4 years ago, and never), and having a family physician^13^. Patients’ questioning attitude was measured by asking, “How would you rate how well you were able to ask your physician about something you did not understand in the last visit?” (1 = poor, 2 = fair, 3 = good, and 4 = excellent)^13^. Health status covariates consisted of high-risk diseases associated with the severity of influenza and pneumonia (stroke, heart disease, diabetes, respiratory disease, kidney/prostate disease, or blood/immune disease)^19,20^, history of influenza or pneumonia in the past year, depressive symptoms (the short form of the geriatric depression scale: not depressed, depressive tendency, or depression)^21^, and homeboundness (going to the field or immediate neighborhood, for shopping, to the hospital, etc.: once a week and more or less). Social relationship covariates consisted of civic participation, social cohesion, and reciprocity^7,22–24^.

### Statistical analyses

We created the Directed Acyclic Graph (DAG) to identify a Minimal Sufficient Adjustment Sets (MSAS) of potential confounders to estimate a direct effect of competence on influenza and pneumococcal vaccinations among older adults. We used an online tool, DAGitty v3.0 (http://www.dagitty.net) to create the DAG. The DAG analyses revealed that the MSAS to estimate the effects were age, sex, marital status, educational attainment, equivalized income, household structure, smoking status, self-rated health, medical checkup, family physician, patient’s questioning attitude, high-risk disease, pneumococcal or influenza vaccination, history of influenza or pneumonia, geriatric depression, homeboundness, civic participation, social cohesion, and reciprocity. We generated 20 imputed data sets for handling missing data by using the chained equations method^25^. Chi-squared tests were used for categorical variables. A Poisson regression model was used to calculate the incident rate ratio (IRR) and 95% confidence interval (CI) based on robust standard errors for the association between the incapability of activities and non-receipt of vaccinations because the prevalence of no vaccination exceeded 10%^26,27^. We carried out sensitivity analyses with the complete data set without missing data as conducted in the imputed data sets. All P values were two-tailed and the significance was set at 5%. We used Stata version 17.1 (Lightstone Corp., College Station, TX, USA) for all analyses.

### Ethical consideration

The process of obtaining informed consent in the present study was as follows: the questionnaire was sent by mail with the explanation of the study; the participants read the written explanation about the purpose of study and replied. Hence, we considered that informed consent was provided by those who replied and sent back the questionnaire. The JAGES protocol in 2019 was approved by the ethics committee of National Center for Geriatrics and Gerontology (No. 992), the ethics committee of Chiba University (No. 2493), and JAGES (No. 2019-01). We followed the STROBE Statement to report our observational study. This study was performed in accordance with the principles of the Declaration of Helsinki. Informed consent was obtained from all participants.

### Institutional review board statement

The study was conducted according to the guidelines of the Declaration of Helsinki. The JAGES Committee (no. 2019-01), the National Center for Geriatrics and Gerontology (no. 1274-2), and the Chiba University Ethics Committee (no. 3442) approved the parent JAGES protocol.

## Results

Table 1 shows the variables of participants by the status of influenza, pneumococcal, and the both of the two vaccinations. A  percentage of older adults with normal capability in the IA or SR who received influenza vaccination was higher than those who did not receive the vaccination, while the percentage of those with normal or the lower capability in the IADL or TMIG-IC with the vaccination was higher than those without it. Older adults with the following characteristics were more likely to receive influenza vaccination than their counterparts: aged ≥ 75 years, being female, being widowed, had education for > 6 or 6–9 years, had < 0.5 or ≥ 4.00 million yen of the equivalized income, lived with offspring, lived in a three-generation household, or lived in households other than the five, never smoked, rated their health status as “good”, “fair”, or “poor,” had a medical checkup within a year, had a family physician, rated their questioning attitude on a physician visit as “excellent,” had  ≥1 high-risk disease that potentially exacerbates influenza or pneumonia, received pneumococcal vaccination, had a history of influenza or pneumonia in the past year, was not depressed, was homebound, and had civic participation, social cohesion, or reciprocity.  A percentage of older adults with normal capability in the IADL, IA, or SR who received pneumococcal vaccination was higher than those without the vaccination, while the percentage of those with normal or the lower capability in the TMIG-IC with the vaccination was higher than those without it. Those with the following characteristics were more likely to receive pneumococcal vaccination than their counterparts: aged ≥ 75 years, being female, being married or widowed, had education for 6–9 or ≥13 years, had 1.00–1.99 or ≥ 4.00 million yen of the equivalized income, lived with a spouse or offspring, lived in a three-generation household, quit smoking ≥ 5 years ago or never smoked, rated their health status as “good”, had a medical checkup within a year, had a family physician, rated their questioning attitude on a physician visit as “excellent,” had  ≥1 high-risk disease, received influenza vaccination, had a history of influenza or pneumonia in the past year, was not depressed, was not homebound, and had civic participation, social cohesion, or reciprocity. A percentage of older adults with normal capability in the IADL, IA, or SR who received either of the two vaccinations was higher than those who received neither of the two vaccinations, while the percentage of those with either of the two was higher than those with neither of them. Those with the following characteristics were more likely to receive either influenza or pneumococcal vaccination than their counterparts: aged ≥ 75 years, being female, being married or widowed, had education for > 6 years, 6–9 years, had ≥ 4.00 million yen of the equivalized income, lived with spouse or offspring, lived in a three-generation household, quit smoking ≥ 5 years ago or never smoked, rated their health status as “good”, “fair”, or “poor”, had a medical checkup within a year, had a family physician, rated their questioning attitude on a physician visit as “excellent,” had  ≥1 high-risk disease a, had a history of influenza or pneumonia in the past year, was not depressed, was not homebound, and had civic participation, social cohesion, or reciprocity.

**Table 1:** Variables of participants by the status of influenza or pneumococcal vaccinations.

		Vaccination											
		Influenza				Pneumococcal				Either (E) or neither (N)			
		Yes	SD	No	SD	Yes	SD	No	SD	E	SD	N	SD
IADL	0	90.7	0.23	91.3	0.29	91.5	0.23	90.2	0.30	91.1	0.20	90.6	0.37
	1*	6.4	0.19	5.8	0.24	6.0	0.19	6.5	0.24	6.2	0.17	6.2	0.31
	2**	2.9	0.13	2.9	0.17	2.6	0.13	3.3	0.18	2.8	0.12	3.2	0.22
Intellectual activity	0	67.0	0.37	61.8	0.51	67.5	0.38	61.5	0.49	66.8	0.34	59.8	0.63
	1	22.4	0.33	25.2	0.45	22.5	0.34	24.8	0.43	22.7	0.30	25.6	0.56
	2	10.6	0.24	13.0	0.35	10.0	0.25	13.7	0.34	10.4	0.22	14.6	0.45
Social role	0	50.3	0.40	43.4	0.53	50.0	0.41	44.4	0.51	49.6	0.36	42.2	0.65
	1	26.1	0.35	27.5	0.47	26.5	0.36	26.7	0.45	26.4	0.32	27.1	0.58
	2	23.6	0.33	29.2	0.48	23.5	0.34	28.8	0.46	24.0	0.31	30.6	0.59
TMIG-IC	0	38.6	0.39	32.5	0.49	38.6	0.41	33.1	0.47	38.1	0.36	31.0	0.60
	1	25.2	0.35	24.5	0.46	25.4	0.37	24.3	0.43	25.3	0.32	24.0	0.55
	2	36.2	0.38	43.1	0.53	36.0	0.40	42.6	0.50	36.6	0.35	45.0	0.65
Age (years)	65–74	46.8	0.39	62.2	0.50	49.1	0.40	57.3	0.49	49.4	0.36	61.5	0.62
	≥ 75	53.2	0.39	37.8	0.50	50.9	0.40	42.7	0.49	50.6	0.36	38.5	0.62
Sex	Male	44.7	0.39	53.0	0.52	44.9	0.40	52.0	0.50	45.6	0.35	54.4	0.63
	Female	55.3	0.39	47.0	0.52	55.1	0.40	48.0	0.50	54.4	0.35	45.6	0.63
Marital status	Married	72.6	0.35	72.9	0.47	73.3	0.36	71.9	0.45	73.2	0.32	71.3	0.58
	Widowed	20.3	0.32	16.3	0.39	19.5	0.32	17.8	0.38	19.4	0.28	16.9	0.48
	Divorced	4.0	0.16	5.8	0.25	4.1	0.16	5.5	0.23	4.2	0.14	6.2	0.31
	Never married	2.4	0.12	4.1	0.21	2.3	0.12	4.0	0.19	2.5	0.11	4.6	0.27
	Other	0.7	0.07	0.8	0.10	0.7	0.07	0.9	0.09	0.7	0.06	1.0	0.13
Educational attainment	< 6	1.0	0.09	0.7	0.09	0.9	0.08	0.9	0.10	0.9	0.08	0.8	0.12
	6–9	28.7	0.36	22.7	0.45	27.3	0.36	25.4	0.44	27.4	0.32	23.8	0.55
	10–12	41.8	0.39	43.3	0.52	42.3	0.40	42.3	0.50	42.3	0.36	42.5	0.63
	≥ 13	27.8	0.35	32.6	0.49	28.8	0.36	30.6	0.45	28.6	0.32	32.2	0.59
	Others	0.8	0.07	0.8	0.09	0.8	0.07	0.8	0.09	0.8	0.06	0.8	0.11
Equivalized income, million yen	< 0.5	11.8	0.25	11.1	0.33	11.2	0.26	12.0	0.33	11.4	0.23	12.0	0.41
	0.50–0.99	30.0	0.35	32.7	0.49	30.2	0.37	32.1	0.46	30.3	0.33	33.1	0.60
	1.00–1.99	19.6	0.31	20.5	0.42	20.1	0.32	19.7	0.39	19.9	0.28	20.0	0.51
	2.00–3.99	13.7	0.27	14.9	0.37	14.1	0.28	14.1	0.34	14.1	0.25	14.1	0.44
	≥ 4.00	24.9	0.34	20.9	0.42	24.3	0.35	22.2	0.41	24.3	0.30	20.9	0.52
Household structure (living with others or alone)	A spouse	49.8	0.39	51.0	0.53	50.6	0.41	49.7	0.50	50.3	0.36	50.2	0.64
	Alone	15.0	0.29	16.4	0.39	14.7	0.30	16.6	0.38	14.8	0.26	17.6	0.49
	Offspring	7.7	0.22	7.0	0.27	7.6	0.22	7.2	0.27	7.6	0.20	6.9	0.33
	A spouse and offspring	8.1	0.21	9.4	0.31	8.5	0.23	8.7	0.28	8.5	0.20	8.8	0.36
	Three-generation household	10.6	0.24	7.6	0.28	10.2	0.24	8.5	0.28	10.1	0.22	7.7	0.34
	Other than described above	8.9	0.22	8.5	0.29	8.4	0.22	9.3	0.29	8.7	0.20	8.8	0.36
Smoking status	Smoking almost everyday	6.3	0.19	12.3	0.34	6.2	0.19	11.9	0.32	6.8	0.18	13.8	0.44
	Smoking sometimes	1.5	0.10	1.8	0.14	1.4	0.10	1.9	0.14	1.4	0.09	2.0	0.18
	Quit smoking < 5 years ago	3.2	0.14	4.4	0.22	3.2	0.15	4.2	0.20	3.3	0.13	4.6	0.27
	Quit smoking ≥ 5 years ago	27.1	0.35	28.5	0.47	27.7	0.37	27.5	0.44	27.6	0.32	27.5	0.57
	Never smoked	61.9	0.38	53.0	0.52	61.5	0.40	54.5	0.49	60.8	0.35	52.0	0.63
Self-rated health	Excellent	13.3	0.27	16.0	0.39	13.7	0.29	15.2	0.36	13.7	0.25	16.3	0.47
	Good	72.1	0.35	71.1	0.48	72.5	0.37	70.6	0.46	72.3	0.33	70.2	0.59
	Fair	12.8	0.26	11.4	0.33	12.1	0.26	12.5	0.33	12.4	0.24	11.9	0.41
	Poor	1.8	0.10	1.5	0.13	1.6	0.10	1.8	0.13	1.7	0.09	1.6	0.16
Medical checkup	Within a year	67.5	0.37	55.8	0.52	68.7	0.38	55.2	0.49	67.4	0.34	50.5	0.63
	1–4 years ago	13.1	0.27	15.4	0.38	13.0	0.28	15.3	0.36	13.3	0.25	16.1	0.47
	> 4 years ago	8.0	0.22	11.8	0.34	7.9	0.23	11.6	0.32	8.1	0.20	13.2	0.43
	Never	11.3	0.25	17.0	0.39	10.4	0.25	17.9	0.38	11.2	0.23	20.2	0.51
Family physician	No	12.7	0.29	29.5	0.48	14.4	0.29	25.3	0.44	14.4	0.27	32.4	0.60
	Yes	87.3	0.29	70.5	0.48	85.6	0.29	74.7	0.44	85.6	0.27	67.6	0.60
Patients’ questioning attitude	Excellent	66.3	0.40	60.6	0.54	66.1	0.42	61.4	0.50	65.7	0.37	59.7	0.65
	Good	22.2	0.36	24.2	0.49	22.2	0.38	23.9	0.45	22.4	0.33	24.5	0.59
	Fair	6.9	0.20	9.3	0.32	7.0	0.22	8.9	0.29	7.2	0.19	9.5	0.39
	Poor	4.7	0.17	5.9	0.25	4.7	0.17	5.8	0.23	4.7	0.15	6.3	0.31
High-risk disease	No	69.5	0.36	74.0	0.46	70.0	0.37	72.7	0.44	70.1	0.33	74.3	0.56
	≥ 1	30.5	0.36	26.0	0.46	30.0	0.37	27.3	0.44	29.9	0.33	25.7	0.56
Pneumonia or influenza vaccination	No	24.5	0.35	32.9	0.50	19.7	0.33	60.6	0.49	-	-	-	-
	Yes	75.5	0.35	67.1	0.50	80.3	0.33	39.4	0.49	-	-	-	-
History of influenza or pneumonia in the past year	No	79.3	0.34	87.9	0.37	80.4	0.37	85.3	0.41	80.5	0.31	88.3	0.44
	Yes	20.7	0.34	12.1	0.37	19.6	0.37	14.7	0.41	19.5	0.31	11.7	0.44
Depression	Not applicable	79.1	0.34	77.0	0.46	80.0	0.34	75.9	0.44	79.3	0.31	75.4	0.58
	Homeboundness	16.7	0.31	18.0	0.42	16.2	0.32	18.7	0.40	16.5	0.29	19.2	0.53
	Depression	4.2	0.16	5.0	0.23	3.8	0.16	5.4	0.22	4.2	0.14	5.4	0.29
Homeboundness	No	96.2	0.15	96.4	0.19	96.4	0.15	96.1	0.19	96.3	0.13	96.1	0.25
	Homeboundness	3.8	0.15	3.6	0.19	3.6	0.15	3.9	0.19	3.7	0.13	3.9	0.25
Social participation	No participation	62.2	0.40	63.5	0.52	60.9	0.41	65.4	0.49	61.6	0.37	65.8	0.62
	≥ 1	37.8	0.40	36.5	0.52	39.1	0.41	34.6	0.49	38.4	0.37	34.2	0.62
Social cohesion	No social cohesion	14.3	0.28	16.1	0.39	14.3	0.29	15.9	0.38	14.4	0.26	16.8	0.48
	≥ 1	85.7	0.28	83.9	0.39	85.7	0.29	84.1	0.38	85.6	0.26	83.2	0.48
Reciprocity	No reciprocity	3.4	0.16	3.5	0.20	3.3	0.16	3.6	0.21	3.2	0.14	3.9	0.26
	≥ 1	96.6	0.16	96.5	0.20	96.7	0.16	96.4	0.21	96.8	0.14	96.1	0.26

*IADL* instrumental activity of daily life, *IA* intellectual activity, *SR* social role, *TMIG-IC* Tokyo Metropolitan Institute of Gerontology Index of Competence.

*Incapable of any one task/activity.

**Incapable of any two tasks/activities.

Table 2 shows the IRRs and 95% CIs for non-receipt of influenza, pneumonia, or the both vaccinations after adjusting for all covariates. Incapability of IA, SR, or TMIG-IC was positively associated with non-receipt of influenza vaccination (IA: for one incapable task/activity: IRR 1.05, 95% CI 1.02–1.09; SR: for one incapable task/activity: IRR 1.08, 95% CI 1.04–1.12; for two incapable tasks/activities: IRR 1.12, 95% CI 1.08–1.16; TMIG-IC: for two incapable tasks/activities: IRR 1.14, 95% CI 1.08–1.21). Unexpectedly, incapability of IADL was negatively associated with influenza vaccination (one incapable task/activity: IRR 0.93, 95% CI 0.87–0.99) Incapability of IADL or IA was positively associated with non-receipt of pneumococcal vaccination (IADL: for two incapable tasks/activities: IRR 1.13, 95% CI 1.05–1.23; IA: for two incapable tasks/activities: IRR 1.10, 95% CI 1.08–1.12). Incapability of IADL, IA, SR, or TMIG-IC was positively associated with non-receipt of the both vaccinations (IADL: for two incapable tasks/activities: IRR 1.17, 95% CI 1.03–1.33; IA: for one incapable task/activity: IRR 1.07, 95% CI 1.02–1.13 and for two incapable tasks/activities: IRR 1.18, 95% CI 1.11–1.25; SR: for one incapable task/activity: IRR: 1.06, 95% CI 1.01–1.12 and for two incapable tasks/activities: IRR 1.13, 95% CI 1.07–1.20; TMIG-IC: for two incapable tasks/activities: IRR 1.14, 95% CI 1.08–1.21). The result of sensitivity analysis with the complete data set without imputation was almost matched with the result analyzed with the imputed data sets (Supplemental Table 2).

**Table 2:** IRRs and 95% CIs of associations between non-receipt of vaccinations and IADL, IA, SR, or TMIG-IC.

	Number of incapable tasks/activities	Influenza vaccination	Pneumococcal vaccination	Neither of the two vaccinations
	0	1.00 (Reference)	1.00 (Reference)	1.00 (Reference)
IADL	1*	0.93 (0.87–0.99)	1.04 (0.98–1.10)	0.97 (0.89–1.07)
	2**	1.04 (0.95–1.14)	1.13 (1.05–1.23)	1.17 (1.03–1.33)
IA	1	1.05 (1.02–1.09)	1.02 (0.99–1.06)	1.07 (1.02–1.13)
	2	1.04 (0.99–1.09)	1.10 (1.08–1.12)	1.18 (1.11–1.25)
SR	1	1.08 (1.04–1.12)	0.99 (0.96–1.03)	1.06 (1.01–1.12)
	2	1.12 (1.08–1.16)	1.02 (0.98–1.06)	1.13 (1.07–1.20)
TMIG-IC	1	1.05 (1.004–1.09)	1.00 (0.96–1.04)	1.05 (0.99–1.12)
	2	1.11 (1.07–1.15)	1.02 (0.99–1.06)	1.14 (1.08–1.21)

*IRR* incident rate ratio, *95% CI* 95% confidence interval, *IADL* instrumental activity of daily life, *IA* intellectual activity, *SR* social role, *TMIG-IC* Tokyo Metropolitan Institute of Gerontology Index of Competence.

*Incapable of any one task/activity.

**Incapable of any two tasks/activities. IRRs and 95% CIs were adjusted for age, sex, marital status, educational attainment, equivalized income, household structure, smoking status, self-rated health, medical checkup, family physician, patient’s questioning attitude, high-risk disease, pneumonia, or influenza vaccination (only for non-receipt of influenza or pneumococcal vaccination), history of influenza or pneumonia, geriatric depression, homeboundness, civic participation, social cohesion, and reciprocity.

Table 3 shows IRRs and 95% CIs of interactions between IADL, IA, SR, or TMIG-IC and a family physician on non-receipt of vaccinations. Having a family physician was negatively associated with non-receipt of influenza vaccination, regardless of incapability in IADL, IA, SR, or TMIG-IC (e.g., IADL: for two incapable tasks/activities: IRR 0.79, 95% CI 0.70–0.88; IA: for two incapable tasks/activities: IRR 0.78, 95% CI 0.73–0.83; SR: for two incapable tasks/activities: IRR 0.83, 95% CI 0.79–0.88; TMIG-IC: for two incapable tasks/activities: IRR 0.82, 95% CI 0.78–0.87). Having a family physician was negatively associated with non-receipt of pneumococcal vaccination, regardless of incapability in IA, SR, or TMIG-IC (e.g., IA: for one incapable task/activity: IRR 0.95, 95% CI 0.90–0.99; SR: for two incapable tasks/activities: IRR 0.94, 95% CI 0.89–0.99; TMIG-IC: for two incapable tasks/activities: IRR 0.93, 95% CI 0.88–0.99). Having a family physician was negatively associated with non-receipt of the both vaccinations, regardless of incapability in  IADL, IA, SR, or TMIG-IC (e.g., IADL: for two incapable tasks/activities: IRR 0.72, 95% CI 0.62–0.84; IA: for two incapable tasks/activities: IRR 0.70, 95% CI 0.64–0.76; SR: for two incapable tasks/activities: IRR 0.69, 95% CI 0.63–0.74; TMIG-IC: for two incapable tasks/activities: IRR 0.68, 95% CI 0.62–0.73). A result of sensitivity analysis with the complete data set without imputation was almost matched with the result analyzed with the imputed data sets (Supplemental Table 3).

**Table 3:** IRRs and 95% CIs of interactions between IADL, IA, SR, or TMIG-IC and a family physician on non-receipt of vaccinations.

	Number of incapable tasks/activities	Home physician	Influenza vaccination	Pneumococcal vaccination	Neither of the two vaccinations
	0	No	1.00 (Reference)	1.00 (Reference)	1.00 (Reference)
IADL	0	Yes	0.73 (0.71–0.76)	0.92 (0.89–0.95)	0.58 (0.56–0.61)
	1*	Yes	0.68 (0.63–0.74)	0.97 (0.90–1.04)	0.59 (0.53–0.67)
	2**	Yes	0.79 (0.70–0.88)	1.07 (0.98–1.17)	0.72 (0.62–0.84)
IA	0	Yes	0.72 (0.69–0.75)	0.91 (0.88–0.95)	0.57 (0.54–0.61)
	1	Yes	0.76 (0.72–0.80)	0.95 (0.90–0.99)	0.63 (0.59–0.68)
	2	Yes	0.78 (0.73–0.83)	0.99 (0.94–1.05)	0.70 (0.64–0.76)
SR	0	Yes	0.74 (0.70–0.77)	0.90 (0.85–0.94)	0.59 (0.54–0.63)
	1	Yes	0.79 (0.75–0.83)	0.89 (0.85–0.94)	0.62 (0.57–0.67)
	2	Yes	0.83 (0.79–0.88)	0.94 (0.89–0.99)	0.69 (0.63–0.74)
TMIG-IC	0	Yes	0.74 (0.69–0.78)	0.89 (0.84–0.95)	0.58 (0.53–0.63)
	1	Yes	0.77 (0.72–0.82)	0.90 (0.84–0.95)	0.60 (0.55–0.66)
	2	Yes	0.82 (0.78–0.87)	0.93 (0.88–0.99)	0.68 (0.62–0.73)

*IRR* incident rate ratio, *95% CI* 95% confidence interval, *IADL* instrumental activity of daily life, *IA* intellectual activity, *SR* social role, *TMIG-IC* Tokyo Metropolitan Institute of Gerontology Index of Competence.

*Incapable of any one task/activity.

**Incapable of any two tasks/activities. The IRRs and 95% CIs were adjusted for age, sex, marital status, educational attainment, equivalized income, household structure, smoking status, self-rated health, medical checkup, family physician, patient’s questioning attitude, high-risk disease, pneumococcal or influenza vaccination (only for non-receipt of influenza or pneumococcal vaccination), history of influenza or pneumonia, geriatric depression, homeboundness, civic participation, social cohesion, and reciprocity.

## Discussion

We investigated the association between lower capacities of higher functions in competency and non-receipt of influenza, pneumococcal, or the both vaccinations among older adults aged 65 years or more. Our results showed that the deterioration of IADL, IA, and SR was positively associated with non-receipt of influenza vaccination, that IADL and IA but not SR was positively associated with pneumococcal vaccination, and that the deterioration of all the three was positively associated with non-receipt of both the vaccinations. In addition, having a family physician mitigated the association between the deterioration of the higher functions and non-receipt of each or the both vaccinations. Our results suggested that maintaining IA and SR are important to undergo the vaccinations according to the recommended schedule and that having a family physician could promote vaccination even for older adults with lower capabilities in functions.

The TMIG-IC assesses IA by asking responders whether they are capable of seeking health-related information by reading articles in the media (see Methods). Moriya et al. showed that IA was associated with general intelligence^28^. This suggests that it is challenging for older adults with lower capabilities in IA to find and understand information related to vaccinations. These older adults may be less likely to receive the vaccinations because they are not interested in and capable of understanding the media’s promotion of the risks and necessity of infectious diseases compared to normal adults. Shar et al. showed that dementia has a negative effect on influenza vaccination among community-dwelling or institutionalized older adults^9^. Our results show that IA is associated with both influenza and pneumococcal vaccinations among community-dwelling older adults. IA in the TMIG-IC predicts dementia risk among functionally independent community-dwelling older adults^29^. Taken together, it is suggested that the lower cognitive capability before dementia may impede vaccination among older adults.

Our results show that SR is associated with two vaccinations among older adults and that the ability to play a SR is also as important for vaccinations among them. To our best knowledge, this is the first study that shows the association between SR and vaccinations among older adults. SRs are defined as “social positions that are linked to sets of norms, expectations, rules and behaviors a person is expected to comply with when enacting a role^30^.” SRs may encourage older adults to prevent the spread of infectious diseases as members of society and contribute to the promotion of vaccinations.

We recently showed that there are heterogeneous determinants of influenza and pneumococcal vaccinations among older adults^13^. Our results show that lower capability of SR was significantly associated with non-receipt of influenza vaccination, while the association was weak with non-receipt of pneumococcal vaccination. Nicholls et al. showed motivation to receive influenza vaccination for protecting a family member with a diminished immune system was greater than the one to receive pneumococcal vaccination among adults^31^. SR may be more important to be motivated for receiving influenza vaccination to protect people living together, but it is not so much for receiving pneumococcal vaccination among older adults because of the reason which was discussed above. On the other hand, our results show that lower capability of IADL was associated with non-receipt of pneumococcal vaccination, while it was negatively associated with non-receipt of influenza vaccination, suggesting that IADL is important to receive pneumococcal vaccination than influenza vaccination and that the lower capability enhances influenza vaccination, which is opposite to our hypothesis. There may be difference in accessibility of pneumococcal and influenza vaccinations for older adults with the lower capability of IADL because there is a difference between the two vaccinations in implemented year as the national program: the program for influenza vaccination started since 2001, while the one for pneumococcal vaccination did since 2014^7,32^. However, further investigation is necessary to examine the hypothesis.

Our results show that incapability of IADL, IA, and SR were associated with non-receipt of both influenza and pneumococcal vaccinations, while IADL or SR and IA were associated with non-receipt of influenza or pneumococcal vaccination. Non-receipt of both the vaccinations was prevalent among older adults. Our results suggest that all the capability of IADL, IA, and SR have an effect on both the vaccinations (24.1%) and that IADL or SR and IA have the same on influenza (16.4%) or pneumococcal vaccination (10.8%).

We and other groups have shown that having a family physician or general practitioner is positively associated with influenza and pneumococcal vaccinations among older adults^13,33,34^. We show that having a family physician mitigated the associations for non-receipt, suggesting that it compensated lower capabilities of IADL, IA, and SR which are important for the vaccinations among older adults. It would be more convenient for older adults with lower capability of IADL to receive vaccinations at the family physician’s clinic in their home town than going to a medical facility far away by using public transport or driving a car independently^35^. It would be easier for those with lower capabilities in IA to understand the risks of the diseases and benefits of the vaccinations, with explanations by their family physician rather than seeking such information themselves^28^. A family physician may increase awareness of the infection risks among older adults who are less likely to recognize the risk of transmission to or from family or friends due to decreased SRs^36,37^.

Our results have some limitations. First, this study has a cross-sectional design, so we did not determine causal pathways. The possibility of a reverse causal relationship between influenza, pneumococcal vaccinations, and competency of older adults potentially cannot be completely excluded; however, it is unlikely that these vaccinations improve lower competency of older adults. Second, the nature of the observational study could not clarify causality because of unmeasured confounders. However, we have tried to adjust for major confounding variables on the individuals and vaccinations. Third, our findings cannot be generalized to people who had been certified as needing long-term care. Fourth, a recall bias may have occurred in the survey for the vaccinations. The impact of this potential bias is unknown.

Vaccinations are necessary to prevent older adults from catching severe influenza and pneumonia. One report among older adults has shown that IADL is associated with these vaccinations. However, it remained uninvestigated whether higher-level functional capacities are associated with the vaccinations. Our results showed that lower capabilities in IA and SR were positively associated with non- receipt of vaccinations as well as IADL and that having a family physician mitigated the associations. Our results suggest that maintaining IA and SR is important for older adults to receive the recommended vaccinations as scheduled and that having a family physician to promote the vaccinations is beneficial even for older adults with limited functions^14–16^. This study indicates that vaccine policies for older adults should consider the higher-level functional capacities and the benefit of having a family physician.

## Supplementary Information

Supplementary Information.

## Data Availability

Data are from the JAGES study. All inquiries are to be addressed to the data management committee via e-mail: dataadmin.ml@jages.net. All JAGES datasets have ethical and legal restrictions on public deposition due to the inclusion of sensitive information from human participants.

## References

[1] Peteranderl C, Herold S, Schmoldt C (2016). Human influenza virus infections. Semin. Respir. Crit. Care Med..

[2] Paget J, Spreeuwenberg P, Charu V (2019). Global mortality associated with seasonal influenza epidemics: New burden estimates and predictors from the GLaMOR Project. J. Glob. Health.

[3] Troeger C, Blacker B, Khalil IA (2018). Estimates of the global, regional, and national morbidity, mortality, and aetiologies of lower respiratory infections in 195 countries, 1990–2016: a systematic analysis for the Global Burden of Disease Study 2016. Lancet Infect. Dis..

[4] Nichol KL, Nordin JD, Nelson DB (2007). Effectiveness of influenza vaccine in the community-dwelling elderly. N. Engl. J. Med..

[5] La J, Km NOY (2003). Effectiveness of pneumococcal polysaccharide vaccine in older adults. N. Engl. J. Med..

[6] Jain A, van Hoek AJ, Boccia D (2017). Lower vaccine uptake amongst older individuals living alone: A systematic review and meta-analysis of social determinants of vaccine uptake. Vaccine.

[7] Kousuke Iwai-Saito, Y. S., Katsunori, K. Social capital and pneumococcal vaccination (PPSV23) in community-dwelling older Japanese: A JAGES multilevel cross-sectional study. *BMJ Open* (2021) in press. 10.1136/bmjopen-2020-043723 PMC8212184 34140341

[8] Chuang YC, Huang YL, Tseng KC (2015). Social capital and health-protective behavior intentions in an influenza pandemic. PLoS ONE.

[9] Shah SM, Carey IM, Harris T (2012). The impact of dementia on influenza vaccination uptake in community and care home residents. Age Ageing.

[10] Lawton, M. P. Assessing the competence of older people. *Research Planning and Action for the Elderly: The Power and Potential of Social Science*, 122–143 (1972).

[11] Manias E, Soh CH, Kabir MZ (2021). Associations between inappropriate medication use and (instrumental) activities of daily living in geriatric rehabilitation inpatients: RESORT study. Aging Clin. Exp. Res..

[12] Fujiwara Y, Shinkai S, Kumagai S (2003). Longitudinal changes in higher-level functional capacity of an older population living in a Japanese urban community. Arch. Gerontol. Geriatr..

[13] Sato K, Kondo N, Murata C (2021). Association of pneumococcal and influenza vaccination with patient-physician communication in older adults: A nationwide cross-sectional study from the JAGES 2016. J. Epidemiol..

[14] Demicheli V, Jefferson T, Di Pietrantonj C (2018). Vaccines for preventing influenza in the elderly. Cochrane Database Syst. Rev..

[15] Q&A on Influenza, Ministry of health labour and welfare, Japan. In *Ministry of Health Labour and Welfare J, ed. Q&A on Influenza*. https://www.mhlw.go.jp/bunya/kenkou/kekkaku-kansenshou01/qa_eng.html

[16] Matanock A, Lee G, Gierke R (2019). Use of 13-valent pneumococcal conjugate vaccine and 23-valent pneumococcal polysaccharide vaccine among adults aged ≥65 years: Updated recommendations of the advisory committee on immunization practices. MMWR Morb. Mortal. Wkly Rep..

[17] Koyano W, Shibata H, Nakazato K (1991). Measurement of competence: Reliability and validity of the TMIG Index of Competence. Arch. Gerontol. Geriatr..

[18] Ministry of health. *Labor and welfare J. Ministry of health. l.a.w., Japan, comprehensive survey of living conditions, 2017* (2017)

[19] Rothberg MB, Haessler SD, Brown RB (2008). Complications of viral influenza. Am. J. Med..

[20] Yndestad A, Damas JK, Oie E (2006). Systemic inflammation in heart failure–the whys and wherefores. Heart Fail. Rev..

[21] Burke WJ, Roccaforte WH, Wengel SP (1991). The short form of the geriatric depression scale: A comparison with the 30-item form. J. Geriatr. Psychiatry Neurol..

[22] Shobugawa Y, Fujiwara T, Tashiro A (2018). Social participation and risk of influenza infection in older adults: A cross-sectional study. BMJ Open.

[23] Saito M, Kondo N, Aida J (2017). Development of an instrument for community-level health related social capital among Japanese older people: The JAGES Project. J. Epidemiol..

[24] Charland KM, Brownstein JS, Verma A (2011). Socio-economic disparities in the burden of seasonal influenza: the effect of social and material deprivation on rates of influenza infection. PLoS ONE.

[25] Royston P, White IR (2011). Multiple imputation by chained equations (MICE): Implementation in Stata. J. Stat. Softw..

[26] Zou G (2004). A modified poisson regression approach to prospective studies with binary data. Am. J. Epidemiol..

[27] Barros AJ, Hirakata VN (2003). Alternatives for logistic regression in cross-sectional studies: An empirical comparison of models that directly estimate the prevalence ratio. BMC Med. Res. Methodol..

[28] Moriya S, Tei K, Miura H (2013). Associations between higher-level competence and general intelligence in community-dwelling older adults. Aging Ment. Health.

[29] Takeda T, Kondo K, Hirai H (2010). Psychosocial risk factors involved in progressive dementia-associated senility among the elderly residing at home. AGES project–three year cohort longitudinal study. Nihon Koshu Eisei Zasshi.

[30] Biddle BJ (1986). Recent developments in role theory. Ann. Rev. Sociol..

[31] Nicholls LAB, Gallant AJ, Cogan N (2021). Older adults' vaccine hesitancy: Psychosocial factors associated with influenza, pneumococcal, and shingles vaccine uptake. Vaccine.

[32] Sugaya N (2014). A review of the indirect protection of younger children and the elderly through a mass influenza vaccination program in Japan. Exp. Rev. Vaccines.

[33] Dominguez A, Soldevila N, Toledo D (2016). Factors associated with pneumococcal polysaccharide vaccination of the elderly in Spain: A cross-sectional study. Hum. Vaccines Immunothe.r.

[34] Ang LW, Cutter J, James L (2017). Factors associated with influenza vaccine uptake in older adults living in the community in Singapore. Epidemiol. Infect.

[35] Okoro CA, Hollis ND, Cyrus AC (2018). Prevalence of disabilities and health care access by disability status and type among adults - United States, 2016. MMWR Morb. Mortal. Wkly Rep..

[36] Higuchi M, Narumoto K, Goto T (2018). Correlation between family physician’s direct advice and pneumococcal vaccination intention and behavior among the elderly in Japan: a cross-sectional study. BMC Fam. Pract..

[37] Bovier PA, Chamot E, Bouvier Gallacchi M (2001). Importance of patients’ perceptions and general practitioners’ recommendations in understanding missed opportunities for immunisations in Swiss adults. Vaccine.

