# Supplementary material for: Association of functional competencies with vaccination among older adults: a JAGES cross-sectional study
Source: Sci Rep. 2022 Oct 14;12:17247. doi: 10.1038/s41598-022-22192-2 (PMC9568573; doi:10.1038/s41598-022-22192-2)
Supplement: Supplementary file 1 — Supplementary Information. [file 41598_2022_22192_MOESM1_ESM.docx]

Supplemental Table 1 Variables of participants by the status of influenza and pneumococcal vaccinations with missing

|  |  | Influenza vaccination | | | | | | Pneumococcal vaccination | | | | | | |
| --- | --- | --- | --- | --- | --- | --- | --- | --- | --- | --- | --- | --- | --- | --- |
|  |  | Yes | | No | | Missing | | Yes | | No | | Missing | | |
|  |  | n | % | n | % | n | % | n | % | n | % | n | | % |
| IADL | 0 | 13,368 | 87.9 | 8,234 | 89.2 | 1,374 | 79.0 | 12,194 | 89.4 | 8,903 | 87.7 | 1,879 | | 78.9 |
|  | 1 | 937 | 6.2 | 528 | 5.7 | 126 | 7.2 | 769 | 5.6 | 647 | 6.4 | 175 | | 7.3 |
|  | 2 | 426 | 2.8 | 264 | 2.9 | 63 | 3.6 | 330 | 2.4 | 334 | 3.3 | 89 | | 3.7 |
|  | Missing | 470 | 3.1 | 210 | 2.3 | 177 | 10.2 | 346 | 2.5 | 273 | 2.7 | 238 | | 10.0 |
| IA | 0 | 9,834 | 64.7 | 5,496 | 59.5 | 860 | 49.4 | 8,986 | 65.9 | 5,999 | 59.1 | 1,205 | | 50.6 |
|  | 1 | 3,255 | 21.4 | 2,238 | 24.2 | 354 | 20.3 | 2,926 | 21.5 | 2,417 | 23.8 | 504 | | 21.2 |
|  | 2 | 1,500 | 9.9 | 1,178 | 12.8 | 291 | 16.7 | 1,229 | 9.0 | 1,361 | 13.4 | 379 | | 15.9 |
|  | Missing | 612 | 4.0 | 324 | 3.5 | 235 | 13.5 | 498 | 3.7 | 380 | 3.7 | 293 | | 12.3 |
| SR | 0 | 7,238 | 47.6 | 3,796 | 41.1 | 698 | 40.1 | 6,528 | 47.9 | 4,272 | 42.1 | 932 | | 39.1 |
|  | 1 | 3,717 | 24.5 | 2,385 | 25.8 | 376 | 21.6 | 3,432 | 25.2 | 2,540 | 25.0 | 506 | | 21.3 |
|  | 2 | 3,498 | 23.0 | 2,643 | 28.6 | 436 | 25.1 | 3,084 | 22.6 | 2,870 | 28.3 | 623 | | 26.2 |
|  | Missing | 748 | 4.9 | 412 | 4.5 | 230 | 13.2 | 595 | 4.4 | 475 | 4.7 | 320 | | 13.4 |
| TMIG-IC | 0 | 5,296 | 34.8 | 2,720 | 29.4 | 423 | 24.3 | 4,845 | 35.5 | 3,018 | 29.7 | 576 | | 24.2 |
|  | 1 | 3,342 | 22.0 | 1,983 | 21.5 | 304 | 17.5 | 3,075 | 22.5 | 2,147 | 21.1 | 407 | | 17.1 |
|  | 2 | 5,206 | 34.2 | 3,800 | 41.1 | 617 | 35.5 | 4,625 | 33.9 | 4,128 | 40.6 | 870 | | 36.5 |
|  | Missing | 1,357 | 8.9 | 733 | 7.9 | 396 | 22.8 | 1,094 | 8.0 | 864 | 8.5 | 528 | | 22.2 |
| Age | 65–74 | 7,220 | 47.5 | 5,761 | 62.4 | 717 | 41.2 | 6,984 | 51.2 | 5,851 | 57.6 | 863 | | 36.2 |
|  | ≥75 | 7,981 | 52.5 | 3,475 | 37.6 | 1,023 | 58.8 | 6,655 | 48.8 | 4,306 | 42.4 | 1,518 | | 63.8 |
| Sex | Male | 6,772 | 44.5 | 4,892 | 53.0 | 822 | 47.2 | 6,138 | 45.0 | 5,276 | 51.9 | 1,072 | | 45.0 |
|  | Female | 8,429 | 55.5 | 4,344 | 47.0 | 918 | 52.8 | 7,501 | 55.0 | 4,881 | 48.1 | 1,309 | | 55.0 |
| Marital status | Married | 10,940 | 72.0 | 6,671 | 72.2 | 1,035 | 59.5 | 10,020 | 73.5 | 7,218 | 71.1 | 1,408 | | 59.1 |
|  | Widowed | 2,975 | 19.6 | 1,476 | 16.0 | 370 | 21.3 | 2,521 | 18.5 | 1,757 | 17.3 | 543 | | 22.8 |
|  | Divorced | 597 | 3.9 | 532 | 5.8 | 80 | 4.6 | 554 | 4.1 | 545 | 5.4 | 110 | | 4.6 |
|  | Never married | 351 | 2.3 | 379 | 4.1 | 53 | 3.0 | 308 | 2.3 | 401 | 3.9 | 74 | | 3.1 |
|  | Other | 96 | 0.6 | 74 | 0.8 | 32 | 1.8 | 75 | 0.5 | 88 | 0.9 | 39 | | 1.6 |
|  | Missing | 242 | 1.6 | 104 | 1.1 | 170 | 9.8 | 161 | 1.2 | 148 | 1.5 | 207 | | 8.7 |
| Educational attainment | <6 | 96 | 0.6 | 50 | 0.5 | 21 | 1.2 | 61 | 0.4 | 75 | 0.7 | 31 | | 1.3 |
|  | 6–9 | 4,030 | 26.5 | 2,027 | 21.9 | 625 | 35.9 | 3,292 | 24.1 | 2,462 | 24.2 | 928 | | 39.0 |
|  | 10–12 | 6,331 | 41.6 | 3,955 | 42.8 | 540 | 31.0 | 5,849 | 42.9 | 4,252 | 41.9 | 725 | | 30.4 |
|  | ≥13 | 4,315 | 28.4 | 3,013 | 32.6 | 333 | 19.1 | 4,124 | 30.2 | 3,119 | 30.7 | 418 | | 17.6 |
|  | Others | 116 | 0.8 | 68 | 0.7 | 13 | 0.7 | 99 | 0.7 | 75 | 0.7 | 23 | | 1.0 |
|  | Missing | 313 | 2.1 | 123 | 1.3 | 208 | 12.0 | 214 | 1.6 | 174 | 1.7 | 256 | | 10.8 |
| Equivalized income, million yen | <0.5 | 1,693 | 11.1 | 1,007 | 10.9 | 311 | 17.9 | 1,373 | 10.1 | 1,196 | 11.8 | 442 | | 18.6 |
|  | 0.50–0.99 | 4,630 | 30.5 | 3,026 | 32.8 | 447 | 25.7 | 4,224 | 31.0 | 3,275 | 32.2 | 604 | | 25.4 |
|  | 1.00–1.99 | 3,085 | 20.3 | 1,901 | 20.6 | 233 | 13.4 | 2,896 | 21.2 | 2,014 | 19.8 | 309 | | 13.0 |
|  | 2.00–3.99 | 2,151 | 14.2 | 1,389 | 15.0 | 155 | 8.9 | 2,061 | 15.1 | 1,447 | 14.2 | 187 | | 7.9 |
|  | ≥4.00 | 3,642 | 24.0 | 1,913 | 20.7 | 594 | 34.1 | 3,085 | 22.6 | 2,225 | 21.9 | 839 | | 35.2 |
|  | Missing | 7,566 | 49.8 | 4,679 | 50.7 | 724 | 41.6 | 6,981 | 51.2 | 5,011 | 49.3 | 977 | | 41.0 |
| Household stricture (living with others or alone) | A spouse | 2,150 | 14.1 | 1,471 | 15.9 | 313 | 18.0 | 1,866 | 13.7 | 1,636 | 16.1 | 432 | | 18.1 |
|  | By alone | 1,063 | 7.0 | 624 | 6.8 | 159 | 9.1 | 934 | 6.8 | 692 | 6.8 | 220 | | 9.2 |
|  | Offspring | 1,206 | 7.9 | 862 | 9.3 | 102 | 5.9 | 1,178 | 8.6 | 864 | 8.5 | 128 | | 5.4 |
|  | A spouse and offspring | 1,622 | 10.7 | 700 | 7.6 | 137 | 7.9 | 1,386 | 10.2 | 851 | 8.4 | 222 | | 9.3 |
|  | Three-generation household | 1,333 | 8.8 | 784 | 8.5 | 155 | 8.9 | 1,123 | 8.2 | 935 | 9.2 | 214 | | 9.0 |
|  | Other than described above | 261 | 1.7 | 116 | 1.3 | 150 | 8.6 | 171 | 1.3 | 168 | 1.7 | 188 | | 7.9 |
|  | Missing | 946 | 6.2 | 1,130 | 12.2 | 138 | 7.9 | 828 | 6.1 | 1,206 | 11.9 | 180 | | 7.6 |
| Smoking status | Smoking almost everyday | 174 | 1.1 | 146 | 1.6 | 44 | 2.5 | 140 | 1.0 | 168 | 1.7 | 56 | | 2.4 |
|  | Smoking sometimes | 394 | 2.6 | 370 | 4.0 | 65 | 3.7 | 370 | 2.7 | 384 | 3.8 | 75 | | 3.1 |
|  | Quit smoking <5 years ago | 4,055 | 26.7 | 2,604 | 28.2 | 407 | 23.4 | 3,772 | 27.7 | 2,756 | 27.1 | 538 | | 22.6 |
|  | Quit smoking ≥5 and more years ago | 9,362 | 61.6 | 4,881 | 52.8 | 965 | 55.5 | 8,307 | 60.9 | 5,513 | 54.3 | 1,388 | | 58.3 |
|  | Never smoked | 270 | 1.8 | 105 | 1.1 | 121 | 7.0 | 222 | 1.6 | 130 | 1.3 | 144 | 6.0 | |
|  | Missing | 1,896 | 12.5 | 1,421 | 15.4 | 193 | 11.1 | 1,784 | 13.1 | 1,474 | 14.5 | 252 | 10.6 | |
| Self-rated health | Excellent | 10,854 | 71.4 | 6,502 | 70.4 | 1,160 | 66.7 | 9,834 | 72.1 | 7,096 | 69.9 | 1,586 | 66.6 | |
|  | Good | 1,894 | 12.5 | 1,042 | 11.3 | 243 | 14.0 | 1,569 | 11.5 | 1,255 | 12.4 | 355 | 14.9 | |
|  | Fair | 258 | 1.7 | 143 | 1.5 | 38 | 2.2 | 212 | 1.6 | 177 | 1.7 | 50 | 2.1 | |
|  | Poor | 229 | 1.5 | 128 | 1.4 | 106 | 6.1 | 240 | 1.8 | 155 | 1.5 | 138 | 5.8 | |
|  | Missing | 10,262 | 67.5 | 5,110 | 55.3 | 830 | 47.7 | 9,506 | 69.7 | 5,554 | 54.7 | 1,142 | 48.0 | |
| Medical checkup | I had one within a year | 1,831 | 12.0 | 1,380 | 14.9 | 235 | 13.5 | 1,635 | 12.0 | 1,490 | 14.7 | 321 | 13.5 | |
|  | I had one sometime between 1 and 4 years ago | 1,147 | 7.5 | 1,064 | 11.5 | 148 | 8.5 | 1,009 | 7.4 | 1,141 | 11.2 | 209 | 8.8 | |
|  | I had one >4 years ago | 1,585 | 10.4 | 1,546 | 16.7 | 350 | 20.1 | 1,238 | 9.1 | 1,778 | 17.5 | 465 | 19.5 | |
|  | I’ve never had one | 376 | 2.5 | 136 | 1.5 | 177 | 10.2 | 251 | 1.8 | 194 | 1.9 | 244 | 10.2 | |
|  | Missing | 1,477 | 9.7 | 2,534 | 27.4 | 240 | 13.8 | 1,616 | 11.8 | 2,352 | 23.2 | 283 | 11.9 | |
| A family physician | No | 13,283 | 87.4 | 6,487 | 70.2 | 1,171 | 67.3 | 11,694 | 85.7 | 7,540 | 74.2 | 1,707 | 71.7 | |
|  | Yes | 441 | 2.9 | 215 | 2.3 | 329 | 18.9 | 329 | 2.4 | 265 | 2.6 | 391 | 16.4 | |
|  | Missing | 8,899 | 58.5 | 4,935 | 53.4 | 738 | 42.4 | 8,071 | 59.2 | 5,491 | 54.1 | 1,010 | 42.4 | |
| Patient questioning attitude | Excellent | 2,979 | 19.6 | 1,920 | 20.8 | 198 | 11.4 | 2,721 | 20.0 | 2,100 | 20.7 | 276 | 11.6 | |
|  | Good | 981 | 6.5 | 785 | 8.5 | 82 | 4.7 | 903 | 6.6 | 834 | 8.2 | 111 | 4.7 | |
|  | Fair | 712 | 4.7 | 541 | 5.9 | 75 | 4.3 | 648 | 4.8 | 579 | 5.7 | 101 | 4.2 | |
|  | Poor | 1,630 | 10.7 | 1,055 | 11.4 | 647 | 37.2 | 1,296 | 9.5 | 1,153 | 11.4 | 883 | 37.1 | |
|  | Missing | 9,995 | 65.8 | 6,381 | 69.1 | 1,036 | 59.5 | 9,069 | 66.5 | 6,915 | 68.1 | 1,428 | 60.0 | |
| High-risk disease | No | 4,595 | 30.2 | 2,386 | 25.8 | 521 | 29.9 | 4,048 | 29.7 | 2,749 | 27.1 | 705 | 29.6 | |
|  | ≥1 | 611 | 4.0 | 469 | 5.1 | 183 | 10.5 | 522 | 3.8 | 493 | 4.9 | 248 | 10.4 | |
|  | Missing | 3,846 | 25.3 | 6,222 | 67.4 | 89 | 5.1 | 2,891 | 21.2 | 6,222 | 61.3 | 123 | 5.2 | |
| History of influenza or pneumonia in the past year | No | 10,611 | 69.8 | 2,891 | 31.3 | 137 | 7.9 | 10,611 | 77.8 | 3,846 | 37.9 | 744 | 31.2 | |
|  | Yes | 744 | 4.9 | 123 | 1.3 | 1,514 | 87.0 | 137 | 1.0 | 89 | 0.9 | 1,514 | 63.6 | |
|  | Missing | 11,623 | 76.5 | 7,814 | 84.6 | 692 | 39.8 | 10,771 | 79.0 | 8,321 | 81.9 | 1,037 | 43.6 | |
| Depression | Not applicable | 1,448 | 9.5 | 557 | 6.0 | 84 | 4.8 | 1,215 | 8.9 | 739 | 7.3 | 135 | 5.7 | |
|  | Homeboundness | 2130 | 14.0 | 865 | 9.4 | 964 | 55.4 | 1,097 | 8.0 | 1,653 | 16.3 | 1,209 | 50.8 | |
|  | Depression | 9,803 | 64.5 | 5,962 | 64.6 | 793 | 45.6 | 9,111 | 66.8 | 6,381 | 62.8 | 1,066 | 44.8 | |
|  | Missing | 2,194 | 14.4 | 1,499 | 16.2 | 243 | 14.0 | 1,900 | 13.9 | 1,688 | 16.6 | 348 | 14.6 | |
| Homeboundness | No | 615 | 4.0 | 453 | 4.9 | 88 | 5.1 | 491 | 3.6 | 538 | 5.3 | 127 | 5.3 | |
|  | Homeboundness | 2589 | 17.0 | 1322 | 14.3 | 616 | 35.4 | 2137 | 15.7 | 1550 | 15.3 | 840 | 35.3 | |
|  | Missing | 14,478 | 95.2 | 8,832 | 95.6 | 1,541 | 88.6 | 13,062 | 95.8 | 9,674 | 95.2 | 2,115 | 88.8 | |
| Social participation | No participation | 539 | 3.5 | 325 | 3.5 | 107 | 6.1 | 428 | 3.1 | 386 | 3.8 | 157 | 6.6 | |
|  | ≥1 | 184 | 1.2 | 79 | 0.9 | 92 | 5.3 | 149 | 1.1 | 97 | 1.0 | 109 | 4.6 | |
|  | Missing | 6,406 | 42.1 | 4,475 | 48.5 | 636 | 36.6 | 5,640 | 41.4 | 4,983 | 49.1 | 894 | 37.5 | |
| Social cohesion | No social cohesion | 5,497 | 36.2 | 3,200 | 34.6 | 352 | 20.2 | 5,284 | 38.7 | 3,346 | 32.9 | 419 | 17.6 | |
|  | ≥1 | 3,298 | 21.7 | 1,561 | 16.9 | 752 | 43.2 | 2,715 | 19.9 | 1,828 | 18.0 | 1,068 | 44.9 | |
|  | Missing | 1,748 | 11.5 | 1,307 | 14.2 | 262 | 15.1 | 1,560 | 11.4 | 1,405 | 13.8 | 352 | 14.8 | |
| Reciprocity | No reciprocity | 13,015 | 85.6 | 7,686 | 83.2 | 1,255 | 72.1 | 11,731 | 86.0 | 8,482 | 83.5 | 1,743 | 73.2 | |
|  | ≥1 | 438 | 2.9 | 243 | 2.6 | 223 | 12.8 | 348 | 2.6 | 270 | 2.7 | 286 | 12.0 | |
|  | Missing | 131 | 0.9 | 168 | 1.8 | 31 | 1.8 | 105 | 0.8 | 181 | 1.8 | 44 | 1.8 | |

IADL: instrumental activity of daily life; IA: intellectual activity; SR: social role; TMIG-IC: Tokyo Metropolitan Institute of Gerontology Index of Competence; * incapable of any one task/activity; ** incapable of any two tasks/activities.

Supplemental Table 2 IRRs and 95% CIs in associations between non-receipt of influenza, pnemococcal, or both vaccinations and IADL, IA, SR, or TMIG-IC in the complete data set

|  | Numbers of incapability of tasks/activities | Influenza  vacination | Pneumococcal  vaccination | Neither of the two vaccinations |
| --- | --- | --- | --- | --- |
| IADL | 0 | 1.00 (Reference) | 1.00 (Reference) | 1.00 (Reference) |
|  | 1* | 0.93 (0.86–1.01) | 1.05 (0.98–1.13) | 0.99 (0.88–1.11) |
|  | 2** | 1.02 (0.90–1.14) | 1.11 (1.001–1.23) | 1.12 (0.95–1.32) |
| IA | 1 | 1.08 (1.03–1.13) | 1.04 (0.996–1.09) | 1.12 (1.04–1.19) |
|  | 2 | 1.06 (0.999–1.13) | 1.13 (1.07–1.19) | 1.22 (1.13–1.33) |
| SR | 1 | 1.05 (1.001–1.10) | 1.00 (0.96–1.05) | 1.04 (0.97–1.12) |
|  | 2 | 1.11 (1.06–1.17) | 1.03 (0.98–1.08) | 1.14 (1.06–1.23) |
| TMIG-IC | 1 | 1.02 (0.96–1.07) | 1.05 (0.99–1.10) | 1.07 (0.99–1.16) |
|  | 2 | 1.11 (1.06–1.17) | 1.04 (0.994–1.09) | 1.17 (1.09–1.26) |

IRR: incident rate ratio; 95% CI: 95% confidence interval; IADL: instrumental activity of daily life; IA: intellectual activity; SR: social role; TMIG-IC: Tokyo Metropolitan Institute of Gerontology Index of Competence; * incapable of any one task/activity; ** incapable of any two tasks/activities. IRRs and 95% CIs were adjusted for age, sex, marital status, educational attainment, equivalized income, household structure, smoking status, self-rated health, medical checkup, family physician, patient’s questioning attitude, high-risk disease, pneumoccoal or influenza vaccination (only for non-receipt of influenza or pneumococcal vaccination), history of influenza or pneumonia, geriatric depression, homeboundness, civic participation, social cohesion, and reciprocity.

Supplemental Table 3 IRRs and 95% CIs of interactions between IADL, IA, SR, or TMIG-IC and a family physician on non-receipt of vaccinations in the complete data

|  | Number of questions with answers “cannot do” | Home physician | Influenza  vacination | Pneumococcal  vaccination | Neither of the two vaccinations |
| --- | --- | --- | --- | --- | --- |
| IADL | 0 | No | 1.00 (Reference) | 1.00 (Reference) | 1.00 (Reference) |
|  | 0 | Yes | 0.73 (0.69–0.76) | 0.92 (0.88–0.96) | 0.60 (0.56–0.64) |
|  | 1* | Yes | 0.69 (0.62–0.76) | 0.98 (0.90–1.08) | 0.62 (0.54–0.72) |
|  | 2** | Yes | 0.74 (0.65–0.85) | 1.05 (0.94–1.18) | 0.70 (0.58–0.85) |
| IA | 0 | Yes | 0.72 (0.68–0.75) | 0.87 (0.81–0.93) | 0.57 (0.53–0.62) |
|  | 1 | Yes | 0.77 (0.72–0.82) | 0.88 (0.82–0.94) | 0.68 (0.61–0.73) |
|  | 2 | Yes | 0.80 (0.74–0.86) | 0.93 (0.87–1.00) | 0.74 (0.66–0.83) |
| SR | 0 | Yes | 0.73 (0.68–0.78) | 0.87 (0.80–0.93) | 0.59 (0.54–0.66) |
|  | 1 | Yes | 0.76 (0.70–0.82) | 0.88 (0.82–0.94) | 0.61 (0.55–0.68) |
|  | 2 | Yes | 0.82 (0.76–0.88) | 0.93 (0.86–1.01) | 0.71 (0.64–0.79) |
| TMIG-IC | 0 | Yes | 0.74 (0.68–0.80) | 0.87 (0.80–0.94) | 0.59 (0.52–0.66) |
|  | 1 | Yes | 0.73 (0.67–0.80) | 0.91 (0.84–0.99) | 0.62 (0.54–0.70) |
|  | 2 | Yes | 0.82 (0.76–0.89) | 0.93 (0.86–1.01) | 0.71 (0.63–0.79) |

IRR: incident rate ratio; 95% CI: 95% confidence interval; IADL: instrumental activity of daily life; IA: intellectual activity; SR: social role; TMIG-IC: Tokyo Metropolitan Institute of Gerontology Index of Competence; * incapable of any one task/activity; ** incapable of any two tasks/activities. IRRs and 95% CIs were adjusted for age, sex, marital status, educational attainment, equivalized income, household structure, smoking status, self-rated health, medical checkup, family physician, patient’s questioning attitude, high-risk disease, pneumoccoal or influenza vaccination (only for non-receipt of influenza or pneumococcal vaccination), history of influenza or pneumonia, geriatric depression, homeboundness, civic participation, social cohesion, and reciprocity.

Supplemental Table 4 Proportion of influenza, pneumonia, or the both vaccinations among the older adults in the imputed data sets (n=26,177)

|  | | Pneumococcal vaccination | |
| --- | --- | --- | --- |
|  |  | Yes | No |
| Influenza  vaccination | Yes | 50.3% | 16.4% |
|  | No | 10.8% | 24.1% |
